# Supplementary material for: Sex‐specific growth and lifespan effects of germline removal in the dioecious nematode Caenorhabditis remanei
Source: Aging Cell. 2024 Jul 31;23(11):e14290. doi: 10.1111/acel.14290 (PMC11561660; doi:10.1111/acel.14290)
Supplement: Supplementary file 1 — Table S1. Table S2. [file ACEL-23-e14290-s001.docx]

**Supplementary materials**

**Sex-specific growth and lifespan effects of germline removal
in the dioecious nematode *Caenorhabditis remanei***

Martin I Lind^1,2^, Brian S Mautz^1,3^, Hanne Carlsson^1,4^, Andrea Hinas^5^, Erik Gudmunds^5,6^ and Alexei A Maklakov^1,4^

^1^ Animal Ecology, Department of Ecology and Genetics, Uppsala University, Uppsala, Sweden

^2^ Department of Environmental and Biosciences, Halmstad University, Halmstad, Sweden

^3^ Population Analytics & Insights, Data Sciences Analytics & Insights, Innovative Medicine Research & Development, Johnson & Johnson, Spring House, PA, USA

^4^ School of Biological Sciences, University of East Anglia, Norwich, UK

^5^ Department of Cell and Molecular Biology, Uppsala University, Uppsala, Sweden

^6^ Evolutionary Biology, Department of Ecology and Genetics, Uppsala University, Uppsala, Sweden

**Table S1**. Virgin lifespan. Median, mean and maximum lifespan as well as number of replicates (n) for each sex and treatment.

| Sex | Treatment | Median lifespan | Mean lifespan | Maximum lifespan | n |
| --- | --- | --- | --- | --- | --- |
| Male | Control | 32 | 32.6 | 61 | 100 |
|  | *glp-1* | 37 | 39.3 | 69 | 99 |
| Female | Control | 34 | 34.4 | 63 | 97 |
|  | *glp-1* | 34 | 34.1 | 67 | 100 |

**Table S2**. Mated lifespan. Median, mean and maximum lifespan as well as number of replicates (n) for each sex and treatment.

| Sex | Treatment | Median lifespan | Mean lifespan | Maximum lifespan | n |
| --- | --- | --- | --- | --- | --- |
| Male | Control | 19 | 18.2 | 24 | 19 |
|  | *glp-1* | 21 | 19.2 | 26 | 9 |
|  | Control + kanamycin | 23 | 23.3 | 37 | 31 |
|  | *glp-1* + kanamycin | 23 | 22.4 | 31 | 37 |
| Female | Control | 16 | 13.7 | 19 | 24 |
|  | *glp-1* | 17 | 12.8 | 24 | 26 |
|  | Control + kanamycin | 13 | 17.5 | 25 | 36 |
|  | *glp-1* + kanamycin | 18 | 18.4 | 33 | 14 |
